# TRUST4RD: tool for reducing uncertainties in the evidence generation for specialised treatments for rare diseases

**DOI:** 10.1186/s13023-020-01370-3

**Published:** 2020-05-26

**Authors:** Lieven Annemans, Amr Makady

**Affiliations:** 1 Dept of Public Health and Primary Care, Faculty of Medicine and Health Sciences, Ghent University, C Heymanslaan 10, 9000 Ghent, Belgium; 2 Dept of Pharmaceutical Sciences, University of Utrecht Faculty David de Wiedbuilding, Universiteitsweg 99, 3584 Utrecht, CG Netherlands

**Keywords:** Evidence generation, Value for money, Health technology assessment (HTA), Orphan medicinal product (OMP), Real world evidence (RWE)

## Abstract

**Background:**

Many treatments developed for rare diseases will have an Orphan Medicinal Product (OMP) designation, indicating that they are likely to deliver benefit in an area of high unmet need. Their approval may be based on a small or uncontrolled trial, as randomised controlled trials (RCTs) of sufficient size are often difficult to conduct, or repeat, as a result of the rarity of the condition, sparsity of patients, or for ethical reasons. Furthermore, many products are given a conditional marketing authorisation, requiring additional evidence to be collected after product launch. This is even more challenging with the advent of advanced therapeutic medicinal products, which use novel scientific approaches like gene or somatic cell therapy.

**Issue:**

Given the high unmet need associated with these products, there is pressure for Health Technology Assessment (HTA)/reimbursement bodies to enable rapid access to effective treatments. However, there is often only limited evidence available for assessment.

**Methods:**

TRUST4RD proposes an approach to identify uncertainties of most concern for decision-makers by developing an iterative and informed dialogue amongst stakeholders (including manufacturers, clinicians, patients, regulatory- and HTA agencies and payers), so that potential approaches to resolution can be discussed. As evidence is generated, uncertainties are reviewed and prioritised, and evidence-generation plans revised or clarified accordingly. The aim is to develop – both pre- and post HTA submission – a better understanding of evidence requirements versus evidence-generation trade-offs as an evidence base grows and the potential value of a product becomes clearer.

**Conclusion:**

TRUST4RD presents guidance on defining uncertainties and evidence gaps in the assessment of value and value for money of specialised treatments for rare diseases. It also provides guidance on the potential of Real World Evidence (RWE) to help address such uncertainties, including the typology of evidence uncertainties, the importance of different uncertainties and the data sources available to address them before and after HTA submission. In making use of the guidance, authorisation and reimbursement discussions on such treatments can be embedded in an evidence-rich context, thereby ensuring value to all parties, particularly to patients.

## Background and objectives

Many of the treatments developed for rare diseases will have an Orphan Medicinal Product (OMP) designation indicating that they are likely to deliver benefit in an area of high unmet need. Their approval may be based on a small or uncontrolled trial, as randomised controlled trials (RCTs) of sufficient size are often difficult to conduct, or to repeat, as a result of the rarity of the condition, sparsity of patients, or because of ethical reasons. Furthermore, many products are given a conditional marketing authorisation, requiring additional evidence to be collected after product launch. This situation has become even more challenging with the advent of advanced therapeutic medicinal products, which use novel scientific approaches like gene therapy, somatic cell therapy or tissue engineered products administered to human beings with a view to regenerating, repairing or replacing a human tissue [1].

Given the high unmet need associated with these products, there is pressure for Health Technology Assessment (HTA)/reimbursement bodies to enable rapid access to effective treatments, which requires spending public money. However, related to the rare and complex nature of the conditions they treat, there is often only limited evidence on these products available for assessment. Uncertainties may occur about the care pathway, natural history, treatments’ clinical outcomes in the longer term, added value to patients and value for money to society [2].

For industry the investment to develop these specialised treatments is important with limited possibilities for economies of scale, and for society there is a high treatment cost per patient, albeit mostly with a rather limited overall budget impact.

As a result of the combination of high prices (albeit for a relatively small number of patients) and uncertainty in evidence, decision-makers in healthcare, particularly HTA agencies and healthcare payers find themselves forced to make decisions about reimbursement under difficult conditions.

Guidance is therefore needed that supports discussion amongst all stakeholders to develop an understanding of the potential evidence that could be generated both pre- and post-HTA submission and to allow trade-offs in different approaches to evidence generation versus the societal issues of access to these medicines and wise use of limited healthcare resources.

This paper aims to develop a technical but pragmatic tool and methodology that allows the uncertainties in evidence for a specialised treatment for a rare disease to be made explicit, to be prioritised and to be addressed in an adequate and timely way. Additionally, the paper provides guidance on the potential of Real World Evidence (RWE) – i.e. data collected outside the context of RCTs – to help address such uncertainties.

It builds on existing initiatives from the European Commission and the European Medicines Agency (EMA) [3–5], the Innovative Medicines Initiative (IMI) [6], the European network for Health Technology Assessment (EUnetHTA) [7], MoCA (Mechanism of Coordinated Access to OMPs) [8], ISPOR [9–11], ORPH-VAL [12], and in papers by Annemans and Pani [13], Hampson et al .[14]., Mitroiu et al. [15] and the EMA post-authorisation efficacy study guidance [16]. Each of these initiatives have tackled one or more issues specifically related to the evidence generation of orphan medicines. For instance, Nestler-Par et al. (2018) provided a taxonomy of impediments to the reseach on and assessment of orphan medicines [10]. Schlander et al. (2016) recommend to assess the effectiveness of orphan medicines differently from the traditional methods and express concerns related to applying the currently prevailing logic of cost-effectiveness to orphan medicines [11]. The current paper aims to set a further step and translate the findings and recommendations from the many exisiting initiatives into a pragmatic and realistic methodology. The proposed tool will provide guidance to inform multi-stakeholder discussions and reimbursement decision-making about specialised treatments for rare diseases. It is aspirational, but builds on the emerging experience of the value of multi-stakeholder dialogues by proposing a structure that explicitly names and addresses uncertainties that are common in specialised treatments for rare diseases.

## Methods

Commissioned by the Belgian Institut National d’Assurance Maladie-lnvalidité/ Rijksinstituut voor ziekte- en invaliditeitsverzekering (INAMI/RIZIV), the paper was developed through a multi-stakeholder dialogue. Starting from a two page document explaining the issues related to evidence uncertainties for orphan medicines, and stating the objectives of the the multi-stakeholder dialogue – namely to develop the methodological tool – stakeholder representatives from patient groups, healthcare professional organisations, government bodies (including payers and HTA authorities) and the pharmaceutical industry were invited to participate in a series of 3 roundtable discussions. During the roundtable sessions, discussions took place under the ‘Chatham House’ rules. After each Roundtable, a summary was written, on which all participants were invited to comment. Initially, existing insights and recommendations from other papers were presented and discussed. During a second roundtable, the components of the tool were established (see below), based on presentations and proposals of the different stakeholders. During a 1 day workshop, template aimed at supporting the development of the tool was pilot tested based on real cases of orphan products for which evidence gaps were observed and whereby the draft tool was applied to systematically address these gaps. During the third workshop, consensus was achieved on the content of the tool. The draft tool was presented at the ISPOR European meeting in Barcelona, novermber 2018 and comments from the audience were taken into account to finalize the tool. A drafting committee comprised of a smaller group of similar stakeholder representatives supported the writing of this paper.

### TRUST4RD components

As stated above, uncertainties exist in the evidence base submitted to HTA for specialised treatments for a rare disease. Uncertainties will, of course, always exist and may also evolve over time. Typically, some uncertainties may be unavoidable at an early stage but may be addressed later on. This paper proposes an approach to identify the uncertainties that are of most concern for decision-makers. It sets out a way to reduce these uncertainties that centres on developing an iterative and informed dialogue amongst stakeholders so that potential approaches to resolve them can be discussed. As evidence is generated, the uncertainties are reviewed and prioritised, so that evidence-generation plans can be revised or clarified. The aim is to develop – both pre- and post HTA submission – a better understanding of evidence requirements versus evidence-generation trade-offs as the evidence base grows and the potential value of a product becomes clearer. Like the advice given in current regulatory and HTA settings, such as Scientific Advice and Early Dialogues, [3, 7] the advices on evidence generation would not be binding on any party.

**Fig. 1:**
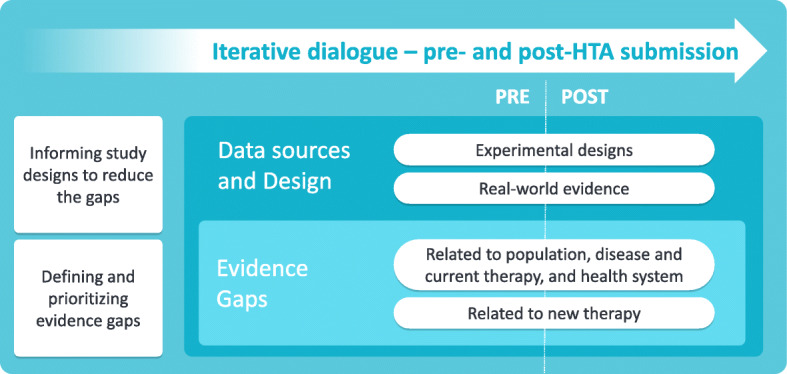
TRUST4RD components

The methodological tool was conceived to consist of three building blocks: evidence gaps, data sources and design, and the presence of an iterative dialogue. Each of these building blocks is subject to an evolution over time, as shown in Fig. 1. The figure also emphasises the distinction between the situation pre- and post HTA, which will be clarified further on in the text. The three building blocks are described in detail below. Naturally, they are not isolated ‘single’ streams but are nested within each other as illustrated in Fig. 1.

### Typology and prioritisation of evidence gaps

We propose a new taxonomy of evidence gaps whereby we distinguish four main types of uncertainties that are common at the time of access decisions for treatments developed for complex or rare diseases: uncertainties related to the size and characteristics of the population; uncertainties related to the natural history of the disease and its current management; uncertainties related to the new treatment; and uncertainties related to the health ecosystem. We describe these here in more detail, using examples, however, without the ambition to provide an exhaustive list. Obviously, for a given orphan medicine multiple uncertainties may exist at the same time.

#### Size and characteristics of the population

The first set of uncertainties relates to the *size and characteristics of the population*. We refer here to uncertainties about the incidence and prevalence of the disease; the exact size of the target population; the characteristics of subpopulations and target populations, such as age and time since diagnosis; the spectrum and variations of disease manifestations, such as symptom severity; and different genotypes or phenoyypes.

#### The natural history of the disease and its current management

The second set of uncertainties is about the *natural history of the disease* and its *current management*. Disease are often characterized by a typical natural course or history of the disease over time, with different prognoses for patients with varying characteristics. In the case of rare diseases evidence with this regard is often lacking. Typically for rare diseases is also the absence of current standards of care, uncertainty about the relevant comparator (might be best supportive care; might also shift over time) [17] as well as the relevant endpoints for clinicians and patients to assess and monitor the disease state. Finally, the extent of the current unmet need, in terms of impact of the disease on quality of life and survival, and the variability herein observed between patients is also subject to uncertainty.

#### The new treatment

The third set of uncertainties is related to the *new treatment*. We refer here to uncertainties about the size of the treatment effect (e.g. large confidence interval resulting from a small sample size); the optimum posology; the treatment effect with different subgroups; the effect size in the real target population when the trial population is different; the characteristics of patients benefiting more from treatment (and the ablity of a biomarker to identify those patients); the durability of the effect and the possibility to retreat after recurrence; the way in which the new treatment will modify further treatments in the treatment sequence; and finally uncertainty about adverse events and safety not yet observed in the trials.

#### The healthcare ecosystem and its actors

Finally, a set of uncertainties is related to the *healthcare ecosystem* and its actors, such as patients, clinicians, and hospitals. We distinguish here uncertainties about patient acceptability and compliance (which are obviously also influenced by the medical need, Quality of Life (QoL) and treatment effect); about prescription patterns (will the medicine be prescribed in the intended patients?); about the available diagnostic and treatment capacity in the healthcare system (will that capacity be sufficient, and which organizational changes will be needed?); about consequences to the healthcare system (for instance: will claimed reductions in hospitalisations also effectively be observed?); and about consequences to society such as reduced absenteeism.

The tool involves as a first step that the known (current and expected) evidence gaps for a given “dossier” are explicitly listed early in the development of the treatment, according to the here described taxonomy .

Obviously, uncertainties and data gaps evolve over time. They might be explained by a combination of factors related to the nature of the disease, its treatment or the system as well as to the quality of the (planned) evidence generation. At the time of submission for HTA some data gaps will be unavoidable (for instance, the long-term effect on survival). Others might however be avoidable if adequate data are obtained during the development process.

The impact of these uncertainties for a given dossier needs to be addressed early to identify the highest priorities for evidence generation. Indeed, some of the uncertainties may have a much larger impact on the eventual value of the treatment than others. The collection of data that are unlikely to help demonstrate the value of a technology should be avoided and therefore a process of prioritisation is needed.

Multi-stakeholder input at this point is essential as stakeholders’ views may differ and coming to a common understanding of issues will help identify priorities. See the first milestone in the iterative dialogue process further on in the text.

An impact score may be envisaged to distinguish between uncertainties that matter most versus those that are much less relevant in the ultimate assessment of value and value for money of the orphan medicine. This score could be informed by both quantitative and qualitative inputs. The quantitative inputs refer to ‘what-if’ scenarios (sensitivity analyses) on key final endpoints such as survival and quality-adjusted survival, cost-offsets etc. The qualitative inputs involve determining the perspectives of patients and clinicians regarding the importance of the uncertainty from their perspective.

### Potential sources to generate reliable evidence

Different data sources/study designs may be needed and can be used (if available) to address the uncertainties in a given dossier and generate reliable evidence. Typically, for the new treatment, some kind of comparative evidence is required [7].

This might not necessarily be a randomized controlled trial. Single arm studies whereby the results are compared to the results in matched historical controls can be a practical and ethically more justified alternative. Prior to or while the evidence generation about the new treatment is undertaken or conducted, several data sources and designs can be applied to reduce the evidence gaps regarding the population, the disease and its current management and the health ecosystem.

Such data can also be collected in an experimental interventional design (for instance an RCT of already existing treatments), or rather based on observation of routine practice in a non-experimental setting, i.e. real- world data that – if well managed, analysed and reported – will lead to real world evidence (RWE).

It is recommended that real-world data about the population, disease, current treatment(s), and health ecosystem can and should be collected as much as possible during the development phase of the new treatment.

Real world evidence about the new treatment can obviously only be collected after market authorisation, with the exception of compassionate use or early access programmes.

Each of these different data sources/designs have inherent pros and cons. We recommend that for each type of important evidence gap, the corresponding data source (experimental or not) and design to reduce that gap is proposed, and that the pros and cons of that data source/design for that specific evidence gap are discussed. Ideally, the most adequate data source/design must be selected to meet the evidence gap in a tailor-made fashion.

### An iterative dialogue and communication line

Finally, uncertainty and increasing understanding between stakeholders requires an iterative communication between a company and the HTA body/payer and regulator for a particular dossier to regularly take stock of newly acquired knowledge and to revise evidence-generation commitments that might not be feasible.

Although we want to emphasise the iterative character of this communication that evolves dependent on the needs of each product, three milestones are identified that we consider to be desirable pre-planned points for each treatment.

A first milestone is an early dialogue between industry and regulatory authorities, such as EMA and HTA/payers, in the presence of clinicians and patient representatives. HTA/payer representatives from different countries should be involved. The rationale is that uncertainties can be avoided or solved by adjusting the design of pivotal interventional studies at an early stage, i.e. before undertaking the pivotal data collection. We refer here, for instance, to the work done by the International Rare Diseases Research Consortium (IRDIRC) on clinical trials with small sample size [18]. It must thereby be made clear, as soon as possible in the development process whether for practical or ethical or other reasons a randomised design is an option or not.

This first dialogue should take place very early in clinical development with all stakeholers to share the scientific ethos behind the development of the treatment, discuss any potential disruptive aspects of the technology in the healthcare system, potential evidence uncertainties and the overall plan for evidence generation [19]. More than one dialogue is, of course, possible during this phase but at least one should be organised. It is obviously also possible to have at this stage bilateral dialogues, i.e. between industry and individual payers, alongside the multi-stakeholder joint dialogue, but the focus at this early stage lies on the latter format. The taxonomy of evidence gaps, and the proposed tailor made data collection must be on the agenda of this early joint dialogue and possible bilateral dialogues.

**Fig. 2:**
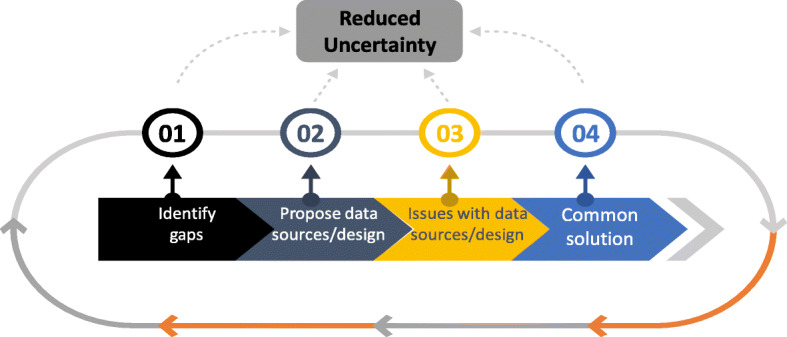
Process for matching data sources/designs with evidence gaps

In each specific dossier, issues will occur with the available/planned data sources and designs. It is important that these issues are explicitly listed and discussed, leading to suggested solutions. The process for this should be as illustrated in Fig. 2.

For Step 1, the list of possible evidence gaps can be consulted to identify the major gaps. For Step 2, the list of available data sources and designs can be consulted. For Step 3, the overview of pros and cons of the different data sources can serve as input. Step 4 requires the application of the principled compromise concept [20, 21]. This concept assumes that during the process and the related debates, statements and communications should be:

- Reliable: there should be no over-claiming;
- Reflective: critically robust positions should be aired and debated;
- Respectful: negotiations should take place in a democratic spirit.

The following example shows how this may work during a first early dialogue:

Example: ① Anticipated gap regarding long-term evidence of the new treatment. ② Plan follow-up of trial patients. ③ This will lead to more information about the sustainability of the effect, but it will still be based on a trial population ➔ ④ A solution might be a timely plan to include patients treated post launch in routine practice in a – preferably existing – registry and observe real-world long-term follow-up outcomes.

Additional issues and solutions were raised during an interactive workshop with industry, representatives from HTA bodies, payers, patient associations and clinicians. We propose that an inventory of issues and solutions is created according to the above framework and regularly updated by HTA authorities so that future submissions and negotiations can learn from these and find inspiration in previous solutions. In the Appendix, examples of such issues and solutions are listed.

Of course, not all uncertainties can be identified and discussed early on. Hence, the early involvement is needed but will not be sufficient to deal with all evidence gaps at that point.

A second dialogue should ideally take place after the completion of the pivotal trial in order to address the still existing uncertainties, with the remaining data gaps explicitly on the agenda. Again, more than one dialogue is possible but at least one should be organised. This dialogue should take place before official submission for HTA and should again involve patients and clinicians. This dialogue may also be bilateral and on a country (or group of countries’) level. Importantly, the potential for post-launch data collection should be discussed here. Indeed, if there is post-launch data collection, this pre-HTA dialogue will already discuss what data should ideally be collected which will facilitate the eventual agreement on post-launch data collection (such an agreement usually takes place after the assessment). The same process as illustrated in Fig. 2 is proposed.

The following example shows how this may work during this pre-HTA dialogue:

Example: ① Uncertainty about the relationship between the effect on a surrogate outcome and the effect on a clinically relevant outcome (e.g. respectively response rate and survival). ② There has been a ‘historical’ correlation shown between the planned surrogate outcome and the clinically relevant outcome, but that was with current standard of care. ③ However, it is likely that this relationship may not be the same for the new treatment ➔ ④ A solution is to accept the historical correlation in the simulations for HTA submission and to explore the new relationship in a registry after launch.

The first and second dialogues are both early dialogues, however, with different timings, namely before and after the major confirmatory evidence on the new therapy. There are existing models for scientific advice/early dialogues in EMA [22] and EUnetHTA [23]. These require organisation and resources from all parties involved with development of a Briefing Book of issues by Industry and a response document by EMA/HTA bodies. During the data-collection period regular submissions are needed to review quality and quantity of data or amend plans as needed.

A third milestone dialogue is then the post-HTA dialogue which will discuss the results of the additional evidence generation after launch and its further consequences. This third milestone is focused on assessing whether the predictions that were made upon the time of HTA submission are validated in the real-world setting. The consequences of ‘not making the promise’ may also be financial if it was decided upon launch to install a risk-sharing agreement. We refer to Makady et al. (2018) for several examples of therapeutic interventions whereby RWE has positively contributed to addressing uncertainties in the evidence base, leading to better informed decision-making [24].

### Necessary conditions

The above explained process, consisting of identifying, classifying and prioritizing evidence gaps, identifying the experimental or non experimental data sources that can be tailored to address the gaps, and the ongoing dialogue with at least 3 contact moments, can only work if certain conditions are put in place.

The first requirement is investing in data generation. Consideration should be given to an EU fund [25] for real-world data generation and supranational collection of data on rare or complex conditions. The European Reference Networks (ERN) seem to be the ideal vehicle for this purpose. Indeed, as the European Commission stated in 2017, no country alone has the knowledge and capacity to treat all rare and complex conditions, but by cooperating and exchanging life-saving knowledge at European level through ERNs, patients across the EU will have access to the best expertise available [26]. We refer to the large list of recommendations issued by Kodra et al. [27] The list includes aspects of governance, Findable, Accessible, Interoperable and Reusable (FAIR) data and information, infrastructure, documentation, training, and quality audit.

With regard to data governance, strong rules are needed to define custodianship of, and access to, data, and to solve issues related to privacy and data integrity. This effort should not be underestimated as it requires investments beyond the building of an infrastructure and consensus on core data elements, endpoints and standardisation.

Moreover, success of the methodological tool described in this paper requires an understanding that to generate evidence for HTA/payers the limitations of data sources and robust analyses are required, but that for some situations the optimal forms of analysis are not yet clear. There needs to be an agreement between the different stakeholders on a specific protocol for data collection but also on the methods used to interpret the results to assess their quality and acceptance by all partners.

Finally, outcomes-based managed-entry agreements between industry and payers may be needed, given the level of uncertainty at the time of launch. These schemes can include individual-based or population-based performance linked reimbursement schemes with money payback guarantee or coverage upon evidence-development schemes, whereby the later reassessment leads to an adaptation of the reimbursement conditions. Despite the potential attractiveness of such schemes, several authors have pointed to the barriers for their implemention [28, 29]. In the earlier mentioned document on *Dynamic outcomes based approaches to pricing and reimbursement of innovative medicines* (Annemans & Pani, February 2017) [13] recognise that there remain concerns – such as administrative burden – about the feasibility of administering these schemes, but also list 10 recommendations for their successful implementation. It is obvious that more investment in better health information systems that reduce administrative burden and improve health data quality is needed.

This tool should lead to more win-win solutions for all stakeholders involved, i.e. allowing payers and policymakers to control expenditure and improve the life of patients, industry to reduce the time lag between marketing authorisation and access to treatment while generating RWE, and patients to have earlier and appropriate access to new treatments.

## Discussion

This paper presents guidance on defining uncertainties and evidence gaps in the assessment of value and value for money of specialised treatments for rare diseases. Additionally, the paper provides guidance on the potential of RWE to help address such uncertainties. In brief, the guidance addresses the following aspects: the typology of evidence uncertainties, the importance of different uncertainties and the (real world) evidence sources available to address such uncertainties either before or after HTA submission. Importantly, this guidance is the result of multi-stakeholder, multi-round discussions and thus combines the different perspectives of stakeholders involved in discussions and decision-making on specialised treatments for rare diseases.

A number of the recommendations made within this guidance correlate well to work within ongoing initiatives. For example, the authors recommend early dialogue between manufacturers, regulatory authorities and HTA agencies on the nature of (real world) evidence to be collected. Presently, EUnetHTA’s work package 5a (WP5a) is specifically addressing this matter. Moreover, the authors advise to continue dialogue in the post-launch setting. Work package 5b (WP5b) of EUnetHTA is addressing the issue of joint post-launch evidence generation (e.g. through patient registries) for HTA purposes. In fact, two pilot projects are ongoing within WP5b, one of which is on a highly specialised treatment.

In the previous section, we briefly refer to additional conditions necessary for advancing the generation of robust (real world) evidence, including structural EU-wide collaboration on data collection, standardisation of databases and data governance. We have also previously mentioned the suitability of the ERNs as a vehicle for this. Another important initiative aiming to address similar issues is the Innovative Medicines Initiative (IMI)-European Health Data Network (EHDN). Over the next few years, the IMI-EHDN will strive to provide a harmonised data infrastructure model and pragmatic governance framework to accommodate cross-centre, cross-country analyses. The outputs of such initiatives are critical to successful implementation of the guidance presented in this article. The principles put forward in the EMA adaptive pathways project (aiming to facilitate best use of existing tools through multi-stakeholder dialogue, including patients), the IMI-ADAPT SMART approach and the New Drug Paradigms (NEWDIGS) also show the important advances already made on this topic.

Clearly, iterative discussions between stakeholders and continuous evidence generation support informed rational evidence generation.

## Conclusion

In conclusion we encourage all stakeholders including manufacturers, clinicians, patients, regulatory and HTA agencies, and payers to make use of the guidance provided in this article as they proceed in developing evidence-generation pathways on specialised treatments for complex or rare conditions. It is our hope that in doing so, authorisation and reimbursement discussions on such treatments can be embedded in an evidence-rich context, thereby ensuring value to all parties, particularly to patients.

## Data Availability

Not applicable.

## Appendix

### Examples of ISSUES and SOLUTIONS

#### Pre- confirmatory trial

① No comparative evidence yet on the new therapy. ② Proposal for conducting an RCT. ③ Randomisation is not possible (argumentation needs to be offered) ➔ ④ A solution might be to design a single-arm study and compare the results with matched historical controls.

① It is anticipated that there will be no long-term data about the new therapy. ② Proposal for collecting data on an intermediate endpoint. ③ There is no good evidence on the relationship between the intermediate endpoint and the long-term clinically relevant endpoint ➔ ④ A solution might be to undertake parallel research on the disease and its current therapy to better understand the relationship between intermediate endpoint(s)/biomarkers and clinical endpoint.

① There is an anticipated gap regarding long-term evidence of the new treatment. ② Proposal to plan follow-up of trial patients. ③ This will lead to more information about the sustainability of the effect, but it will still be based on a trial population ➔ ④ A solution might be a timely plan to include patients treated post launch in routine practice in a – preferably existing – registry and observe real-world, long-term follow-up outcomes.

① The unmet need in terms of quality of life of patients is unclear. ② Proposal to collect baseline data on QoL in the trial. ③ There is concern about patient selection for the trial ➔ ④ A solution might be to assess QoL in all patients per centre, whether or not participating in the trial.

① For ethical reasons a traditional RCT is not possible. ② A ‘delayed start’ or crossover design is proposed; ③ This means that at the end of the trial, all patients will be treated with the new treatment ➔ ④ A solution might be to compare outcomes of the ‘early starters’ and the ‘delayed starters’ with a matched historical cohort.

#### Pre-HTA, after confirmatory trial

① By the time of submission, there will still be uncertainty about the relationship between the effect on a surrogate outcome and the effect on a clinically relevant outcome (e.g. respectively, response rate and survival) ② There has been a ‘historical’ correlation shown between the planned surrogate outcome and the clinically relevant outcome, but that was with current standard of care. ③ However, it is likely that this relationship may not be the same for the new treatment ➔ ④ A solution is to accept the historical correlation in the simulations for HTA submission and to explore the new relationship in a registry post launch.

① By the time of submission, the number of expected recurrences is uncertain but anticipated to be very low. ②It was proposed to follow up recurrences further on in the trial. ③ The sample size of the trial is too small for this purpose ➔ ④ A solution might be to apply an existing claims database to allow the follow-up of all patients in real life. This could be combined with an outcomes-guarantee contract.

## Abbreviations

ADAPT SMART: Accelerated Development of Appropriate Patient Therapies / Sustainable, Multi-stakeholder Approach from Research to Treatment-outcomes
EHDN: European Health Data Network
EMA: European Medicines Agency
ERN: European Reference Network
EUnetHTA: European Network for Health Technology Assessment
HTA: Health Technology Assessment
IMI: Innovative Medicines Initiative
INAMI: Institut National d’Assurance Maladie-lnvalidité
IRDiRC: International Rare Diseases Research Consortium
ISPE: International Society for Pharmacoepidemiology
ISPOR: International Society for Pharmacoeconomics and Outcomes Research
MoCA: Mechanism of Coordinated Access [to OMPs]
NEWDIGS: NEW Drug Development ParadIGmS
OMP: Orphan Medicinal Product
ORPH-VAL: European Working Group for Value Assessment and Funding Processes in Rare Diseases
PAES: Post-Authorisation Efficacy Studies
PICO: Patients; Intervention; Comparator; Outcomes
QoL: Quality of Life
RIZIV: Rijksinstituut voor ziekte- en invaliditeitsverzekering
RWE: Real World Evidence
